# Postbiotic Sodium Butyrate Mitigates Hypertension and Kidney Dysfunction in Juvenile Rats Exposed to Microplastics

**DOI:** 10.3390/antiox14030276

**Published:** 2025-02-26

**Authors:** You-Lin Tain, Ying-Jui Lin, Chih-Yao Hou, Guo-Ping Chang-Chien, Shu-Fen Lin, Chien-Ning Hsu

**Affiliations:** 1 Division of Pediatric Nephrology, Kaohsiung Chang Gung Memorial Hospital, Kaohsiung 833, Taiwan; tainyl@cgmh.org.tw; 2 College of Medicine, Chang Gung University, Taoyuan 330, Taiwan; 3 Institute for Translational Research in Biomedicine, Kaohsiung Chang Gung Memorial Hospital, Kaohsiung 833, Taiwan; 4 Division of Critical Care, Kaohsiung Chang Gung Memorial Hospital, Kaohsiung 833, Taiwan; rayray@cgmh.org.tw; 5 Division of Cardiology, Kaohsiung Chang Gung Memorial Hospital, Kaohsiung 833, Taiwan; 6 Department of Respiratory Therapy, Kaohsiung Chang Gung Memorial Hospital, Kaohsiung 833, Taiwan; 7 Department of Early Childhood Care and Education, Cheng Shiu University, Kaohsiung 833, Taiwan; 8 Department of Seafood Science, National Kaohsiung University of Science and Technology, Kaohsiung 811, Taiwan; chihyaohou@webmail.nkmu.edu.tw; 9 Institute of Environmental Toxin and Emerging-Contaminant, Cheng Shiu University, Kaohsiung 833, Taiwan; guoping@csu.edu.tw (G.-P.C.-C.); linsufan2003@csu.edu.tw (S.-F.L.); 10 Super Micro Mass Research and Technology Center, Cheng Shiu University, Kaohsiung 833, Taiwan; 11 Center for Environmental Toxin and Emerging-Contaminant Research, Cheng Shiu University, Kaohsiung 833, Taiwan; 12 Department of Pharmacy, Kaohsiung Chang Gung Memorial Hospital, Kaohsiung 833, Taiwan; 13 School of Pharmacy, Kaohsiung Medical University, Kaohsiung 807, Taiwan

**Keywords:** butyrate, oxidative stress, hypertension, gut microbiota, kidney disease, microplastics, inflammation, short-chain fatty acid

## Abstract

Background: Plastic production has led to widespread microplastic (MP) pollution, with children more vulnerable to MPs than adults. However, the mechanisms linking MP exposure to hypertension and kidney disease in children remain unclear. This study explored whether sodium butyrate, a short-chain fatty acid (SCFA) with antioxidant and anti-inflammatory properties, could mitigate MP-induced hypertension and kidney damage in juvenile rats. Methods: Male Sprague-Dawley rats (3 weeks old) were randomly assigned to four groups (n = 8/group): control, low-dose MP (1 mg/L), high-dose MP (10 mg/L), and high-dose MP with sodium butyrate (400 mg/kg/day). Rats were euthanized at 12 weeks. Results: High-dose MP exposure impaired kidney function and increased blood pressure, which were alleviated by sodium butyrate through reduced oxidative stress, modulation of gut microbiota, increased plasma butyric acid levels, and enhanced renal SCFA-sensing G protein-coupled receptor 43 expression. Conclusions: Sodium butyrate holds potential for mitigating MP-induced hypertension by reducing oxidative stress, modulating the gut microbiota, and elevating butyric acid levels.

## 1. Introduction

Environmental pollution poses a significant risk to human health, serving as a major public health concern [1]. It is directly linked to the onset and progression of various chronic diseases, including hypertension and kidney disease [2,3]. Hypertension and kidney disease share a complex, bidirectional relationship, with both conditions capable of originating in childhood [4]. Therefore, early detection and intervention for hypertension and kidney disease in children could play a crucial role in reducing the global health burden.

Plastic production has surged in recent decades, leading to widespread environmental pollution [5]. Accumulated plastics degrade into smaller particles known as microplastics (MPs) and nanoplastics (NPs), which are now found ubiquitously, particularly in the food chain [6]. Recent studies have highlighted that MPs, with polystyrene being the predominant type, can accumulate in the human body, potentially posing a threat to human health [7]. Preclinical animal studies have confirmed that MPs accumulate in various tissues and organs and have linked this accumulation to adverse effects, including hypertension and kidney disease [8,9,10]. MPs have been detected in human kidneys and carotid artery plaques [11,12], suggesting their potential involvement in hypertension and kidney disease. Notably, children are more susceptible to the harmful effects of MPs than adults, as they tend to ingest higher amounts of MPs on a daily basis [13,14].

The proposed mechanisms underlying MP-driven cardiovascular and kidney toxicity include oxidative stress, inflammation, immunomodulation, and apoptosis [9,15]. The dysregulation of the gut microbiome was correlated with hypertension and kidney disease [16,17]. Although gut microbiota dysbiosis has been associated with MP toxicity [18], its role in MP-induced hypertension and kidney disease remains not fully understood. Emerging evidence suggests that manipulating the gut microbiota using probiotics, prebiotics, and postbiotics may help restore microbiome balance and mitigate hypertension and kidney disease [19,20,21].

Postbiotics represent a novel class of biotics, holding significant potential to provide various health benefits [22]. Butyrate, a short-chain fatty acid (SCFA), is produced through microbial fermentation of dietary fibers and exerts its effects on blood pressure (BP) via G protein-coupled SCFA receptors [23]. In addition to regulating BP, butyrate has demonstrated antioxidant and anti-inflammatory properties, functioning as a postbiotic [24]. Sodium butyrate is not only used as a dietary supplement but has also been registered as a drug and is utilized in veterinary medicine. Previously, we reported that sodium butyrate supplementation averted the development of hypertension, accompanied by alterations in gut microbiota [25]. Given the reported toxicity of MPs and the benefits of butyrate administration, we hypothesized that sodium butyrate treatment could protect against MP-induced hypertension and kidney damage in juvenile rats, possibly through mechanisms involving the reduction of oxidative stress, inflammation, and gut microbiota dysbiosis.

## 2. Materials and Methods

### 2.1. Animal Study

The study, approved by the Institutional Animal Ethics Committee at our hospital (permit: 2023090203, 13 March 2024), used male Sprague-Dawley (SD) rats (n = 32) bred in an AAALAC-accredited facility. The progeny were raised with their lactating dams, and the dams had ad libitum access to chow and water during the study. Male juveniles were chosen for their higher early susceptibility to hypertension [26]. After weaning, male offspring (3 weeks old, eight per group) were randomly assigned to four groups: control (CN, given tap water as drinking water), low-dose MP (MPL, given tap water containing 1 mg/L MP), high-dose MP (MPH, given tap water containing 10 mg/L MP), and high-dose MP with sodium butyrate (MPHB, given tap water containing 10 mg/L MP and sodium butyrate). Based on the water intake, the dose of sodium butyrate was adjusted so that the butyrate consumption was 400 mg/kg/day. The sulfate-modified, negatively charged polystyrene MPs (5.0 µm, green color) were custom-designed by Magsphere Inc. (Pasadena, CA, USA; Lot: PSG5638). They were delivered as a 50 mL solution containing 10% solids, with a particle size tolerance of ±10%. To ensure uniform dispersion, the solution was thoroughly agitated using a vortex mixer before being added to the drinking water. Water bottles were cleaned, and fresh solutions were prepared twice weekly. Previous rat studies were used to determine the dosages of MPs and sodium butyrate [25,27]. We did not include a control group treated with sodium butyrate, as it is generally considered safe. Additionally, we focused on investigating the therapeutic effect of sodium butyrate in the high-dose MP-exposed group rather than the low-dose group to minimize the number of animals used in the experiment, in accordance with the 3R principle.

We measured BP every four weeks in trained rats using a tail-cuff sphygmomanometer (CODA, Kent Scientific, Torrington, CT, USA) after one week of acclimation. Urine (24 h) was collected in metabolic cages at 12 weeks prior to sacrifice to measure protein (Bradford method) and creatinine clearance. At 12 weeks, rats were euthanized, and stool samples were stored at −80 °C. One kidney was excised, with the cortex and inner medulla dissected, immediately snap-frozen, and stored at −80 °C. The other kidney was perfusion-fixed for immunohistochemistry. Blood samples were aliquoted and stored at −80 °C, while plasma and urine creatinine levels were analyzed using an Agilent HP 1100 HPLC system (Wilmington, DE, USA).

### 2.2. 8-OHdG Staining

DNA is a primary target of reactive oxygen species (ROS), with hydroxylation of 2′-deoxyguanosine producing 8-hydroxy-2′-deoxyguanosine (8-OHdG), a marker of oxidative DNA damage from environmental exposures [28]. A paraffin-embedded whole kidney section, cut at a thickness of 4 µm, was deparaffinized in xylene, rehydrated through a graded ethanol series, and transferred to phosphate-buffered saline. Immunoreactivity was detected using a primary 8-OHdG antibody (1:100, JaICA, Tokyo, Japan) with the polymer–horseradish peroxidase labeling kit (BIOTnA Biotech, Taipei, Taiwan) and 3,3′-diaminobenzidine as the chromogen. Sections were lightly counterstained with hematoxylin and preserved under cover glass. A negative control was performed by omitting the primary antibody incubation. All sections were stained simultaneously using the same reagents, antibody dilutions, and incubation periods. Positively stained cells in 10 random renal cortex sections were quantified using Ventana Image Viewer 3.2.0.

### 2.3. Cytokine Concentrations in the Serum and Kidneys

As inflammation and hypertension are physiologically inter-related [29], cytokines interleukin (IL)-1A, IL-1B, IL-2, IL-6, and IL-17A, and interferon-γ (IFN-γ) in serum and renal cortical protein extracts were quantified utilizing a LEGENDplex™ multiplex cytokine panel kit (BioLegend, San Diego, CA, USA), following the method described previously [30]. Samples were investigated in duplicate on a BD FACS Canto II flow cytometer (BD Biosciences, San Jose, CA, USA), with cytokine concentrations determined by fluorescence intensity and bead color. Data were analyzed using LEGENDplex software (https://www.biolegend.com/en-ie/immunoassays/legendplex/support/software, accessed on 23 February 2025) (BioLegend, San Diego, CA, USA).

### 2.4. Metagenomic Analysis of the Gut Microbiome

Microbial DNA from rat feces was analyzed via full-length 16S rRNA gene sequencing using PacBio with barcode primers for SMRTbell library preparation (Menlo Park, CA, USA) [10]. For phylogenetic tree construction, QIIME2’s FastTree algorithm was used to analyze amplicon sequence variants (ASVs) [31]. Taxonomic assignments of microbial sequences were performed using the Greengenes reference database. Sequence reads underwent quality filtering to remove low-quality reads, and high-quality reads were screened for chimeras, which were subsequently removed. For α-diversity analysis, we calculated the Pielou and Simpson indices to assess the richness and evenness of microbial communities within each sample. For β-diversity, which measures the differences in microbial composition between groups, we employed partial least squares discriminant analysis (PLSDA) combined with analysis of similarities (ANOSIM). Linear discriminant analysis (LDA) effect size identified bacterial taxa with significant abundance differences between groups [32]. Statistical differences were assessed using a two-sided Welch’s t-test in the statistical analysis of metagenomic profiles (STAMPs).

### 2.5. Analysis of Plasma SCFAs

SCFA concentrations in plasma were analyzed using a gas chromatograph–mass spectrometer (Agilent Technologies, Santa Clara, CA, USA) [25], quantifying acetic, propionic, and butyric acids, as well as smaller amounts of isobutyric, isovaleric, and valeric acids. SCFAs were separated on a DB-FFAP column, with 2-ethylbutyric acid as the internal standard for accurate quantification.

### 2.6. Quantitative PCR

We extracted total RNA from kidney cortex samples and analyzed it using real-time qPCR [10] to assess the expression of SCFA receptors, including G protein-coupled receptors (GPCRs) GPR41, GPR43, GPR109A, and olfactory receptor 78 (Olfr78), with 18S ribosomal RNA used as the reference gene for normalization. Rat-specific sequences were used to design the following forward (FW) and reverse (RV): GPR41 (NC_086019.1) FW 5′-TCTTCACCACCGTCTATCTCAC-3′, and RV 5′-CACAAGTCCTGCCACCCTC-3′; GPR43 (NC_000019.10) FW 5′-CTGCCTGGGATCGTCTGTG-3′, and RV 5′-CATACCCTCGGCCTTCTGG-3′; GPR109A (NC_086030.1) FW 5′-CGGTGGTCTACTATTTCTCC-3′, and RV 5′-CCCCTGGAATACTTCTGATT-3′; Olfr78 (NM_173293) FW 5′-GAGGAAGCTCACTTTTGGTTTGG-3′, and RV 5′-CAGCTTCAATGTCCTTGTCACAG-3′; and R18S (X01117) FW 5′-GCCGCGGTAATTCCAGCTCCA-3′, and RV 5′-CCCGCCCGCTCCCAAGATC-3′. Relative gene expression was calculated using the comparative threshold cycle (Ct) method, with fold changes determined by the formula 2^−ΔΔCt^ to compare target genes to the control.

### 2.7. Statistical Analysis

Data are expressed as the mean ± standard error of the mean (SEM). The Shapiro–Wilk test was used to assess the normality of the data distribution and determine the appropriate statistical approach. If the data followed a normal distribution, differences between groups were analyzed using a one-way analysis of variance (ANOVA), followed by Tukey’s post hoc test for pairwise comparisons. If the data significantly deviated from normality, the non-parametric Kruskal–Wallis test was applied, followed by Dunn’s post hoc test for pairwise comparisons. A *p*-value of less than 0.05 was considered statistically significant. All analyses were performed using SPSS version 17.0 software (SPSS Inc., Chicago, IL, USA).

## 3. Results

### 3.1. Blood Pressure and Kidney Function

MP exposure did not cause mortality in rats at 12 weeks of age, indicating that the exposure was not lethal within the timeframe of the study. Among the four groups, the CN group showed the highest body weight (BW) and kidney weight (KW). However, a significant difference in the ration of KW/BW was observed only between the MPL and MPH groups (Table 1). At 12 weeks of age, systolic BP, diastolic BP, and mean arterial pressure were significantly higher in the MPH group compared with the CN and MPL groups. These increases were attenuated by sodium butyrate therapy in the MPHB group.

**Figure 1:**
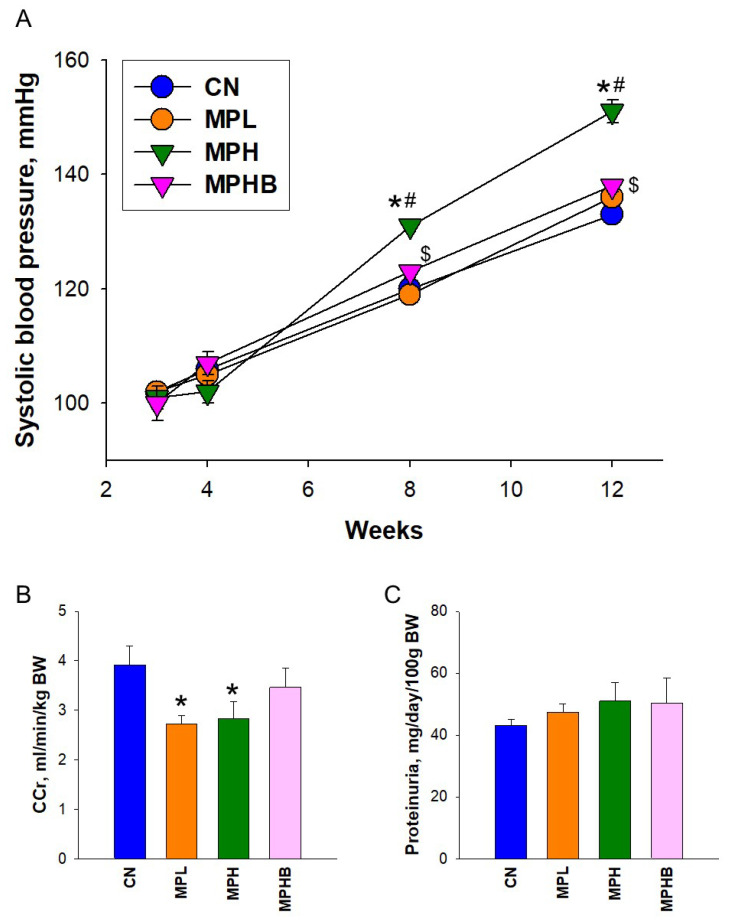
Effect of microplastics and sodium butyrate on blood pressure and kidney outcomes. (**A**) Systolic blood pressure in rats from 3 to 12 weeks of age. (**B**) Creatinine clearance, and (**C**) proteinuria at week 12. Eight per group; * *p* < 0.05 vs. CN; # *p* < 0.05 vs. MPL; $ *p* < 0.05 vs. MPH, indicating that group differences reached statistical significance.

Longitudinal systolic BP measurements in rats at different ages are illustrated in Figure 1A. High-dose MP exposure induced an elevation in systolic BP at 8 and 12 weeks of age, which was mitigated by sodium butyrate treatment. To assess kidney function, we performed the 24 h creatinine clearance (CCr) test, which measures the amount of creatinine in both urine and blood. Both low- and high-dose MP exposures led to impaired kidney function, as indicated by a lower CCr compared to the CN group (Figure 1B).

Although it did not reach statistical significance, a trend was observed in which the reduction of CCr in the MPH group was partially reversed toward control levels by sodium butyrate in the MPHB group. In other words, sodium butyrate treatment partially mitigated the decline in CCr. However, there were no significant differences in 24 h urine excretion, a marker of kidney damage, among the four groups (Figure 1C).

### 3.2. Oxidative Stress

**Figure 2:**
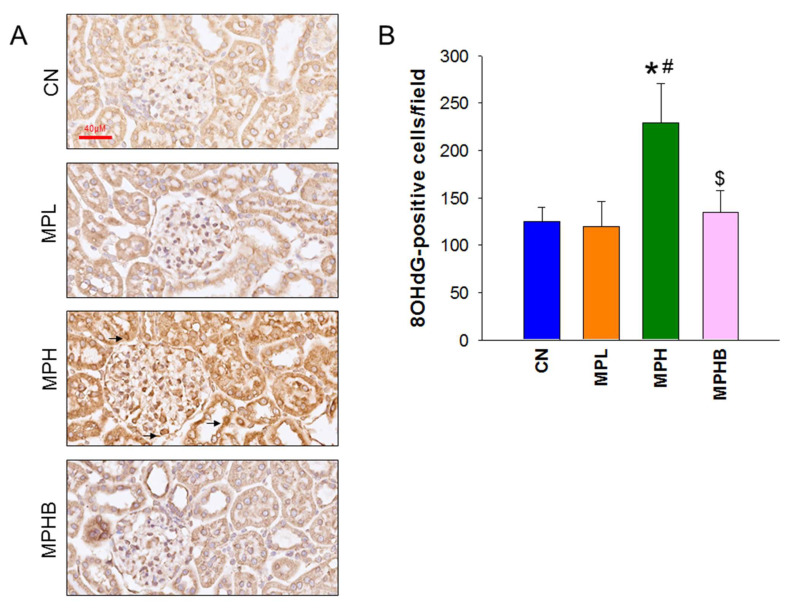
Effect of microplastics and sodium butyrate on oxidative damage in rat kidneys. (**A**) Immunohistochemical staining for 8-OHdG in the kidneys showed its localization in the nuclei of glomeruli and tubular cells (arrows), indicating oxidative damage. (**B**) Quantitative analysis of 8-OHdG-positive cells in the kidney cortex revealed varying levels of oxidative DNA damage. Scale bar = 40 μM. Eight per group; * *p* < 0.05 vs. CN; # *p* < 0.05 vs. MPL; $ *p* < 0.05 vs. MPH, indicating that group differences reached statistical significance.

We then conducted an analysis of 8OHdG immunoreactivity in the kidneys to evaluate the potential connection between oxidative stress, MP-induced hypertension, and kidney damage. As shown in Figure 2, the MPH group revealed the strongest immunoreactivity in glomeruli and tubular cells, with a significant increase in 8OHdG-positive cells compared to the other groups, indicating that sodium butyrate may protect against MP-induced oxidative stress damage.

### 3.3. Inflammation

**Figure 3:**
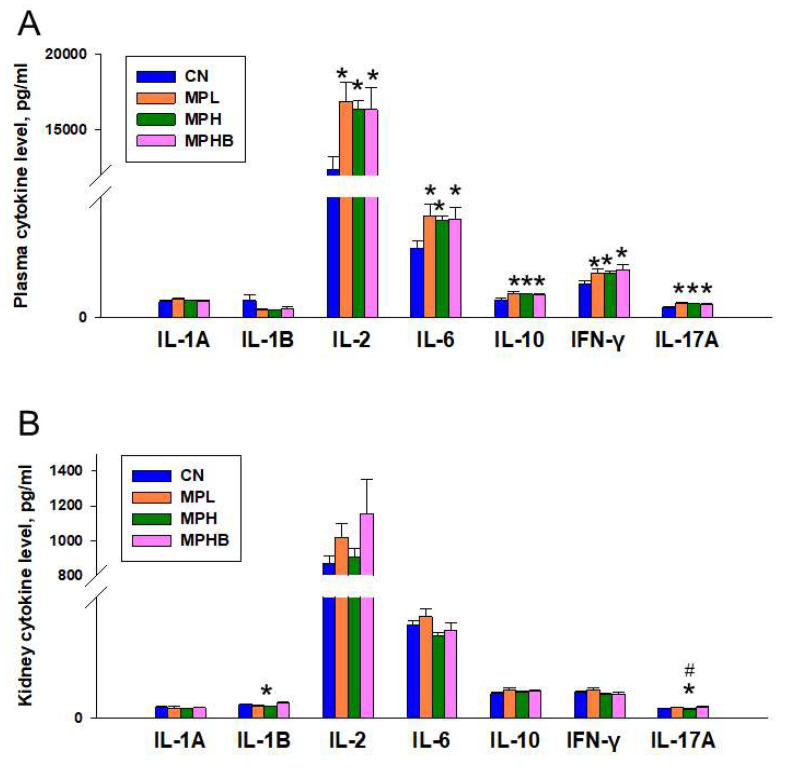
Effect of microplastics and sodium butyrate treatment on cytokine levels in the (**A**) serum and (**B**) kidneys. Eight per group; * *p* < 0.05 vs. CN; # *p* < 0.05 vs. MPL, indicating that group differences reached statistical significance.

We measured the cytokines IL-1A, IL-1B, IL-2, IL-6, IL-10, IL-17A, and IFN-γ in serum and kidney tissues to assess MP-induced inflammation. As shown in Figure 3A, both low- and high-dose MP exposure increased serum IL-2 levels in the MPL (16,907 ± 1234 pg/mL), MPH (16,360 ± 601 pg/mL), and MPHB (16,354 ± 1417 pg/mL) groups compared to the CN group (12,406 ± 862 pg/mL). Similarly, the CN group exhibited the lowest plasma concentrations of IL-6 (749 ± 75.4 pg/mL), IL-10 (193.7 ± 14.1 pg/mL), IFN-γ (364.7 ± 33.5 pg/mL), and IL-17A (108.9 ± 7.8 pg/mL) among the four groups. Renal IL-1B and IL-17A levels were lower in the MPH group (IL-1B: 9.65 ± 0.47 pg/mL; IL-17A: 7.68 ± 0.23 pg/mL) than in the CN group (IL-1B: 11.53 ± 0.42 pg/mL; IL-17A: 8.5 ± 0.17 pg/mL) (Figure 3B).

### 3.4. Gut Microbiota Composition

**Figure 4:**
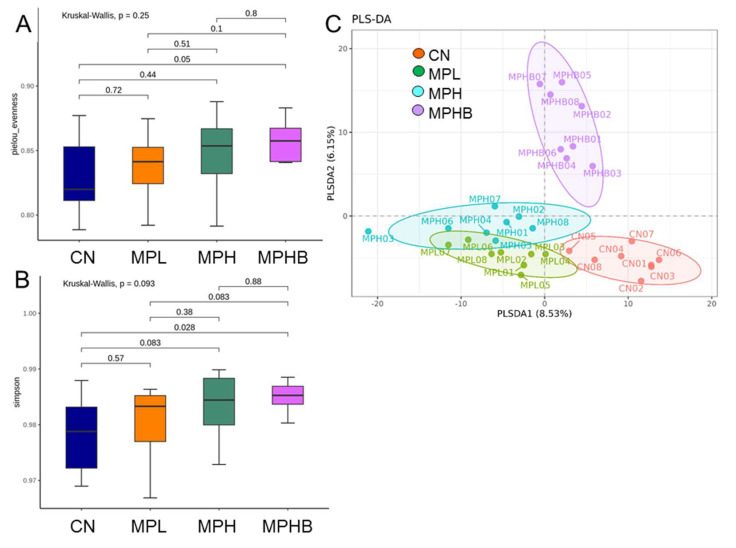
Effect of microplastics and sodium butyrate on gut microbiota composition in 12 week-old rats. α-diversity and β-diversity among groups: (**A**) Pielou index, (**B**) Simpson index, and (**C**) partial least squares discriminant analysis (PLSDA) was used to visualize β-diversity. Each point represents an individual sample’s microbiota, color-coded by group assignment. Eight per group.

We assessed two α-diversity metrics, the Pielou index (Figure 4A) and Simpson index (Figure 4B), to estimate microbiome evenness and richness. Our analysis showed no significant differences in the Pielou index (*p* = 0.25) or the Simpson index (*p* = 0.093). However, sodium butyrate treatment resulted in lower Pielou (*p* < 0.05) and Simpson (*p* = 0.028) values than those in the CN group (Figure 4B). For β-diversity, a PLSDA plot was generated, showing four distinct clusters (Figure 4C). ANOSIM analysis further established significant differences between most groups, except for the comparison between the MPL and MPH groups (*p* = 0.059) and between the MPH and MPHB groups (*p* = 0.085).

**Figure 5:**
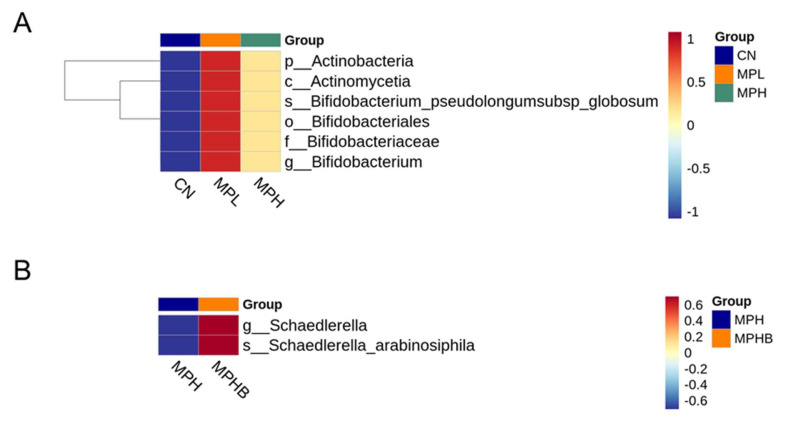
Effect of microplastics and sodium butyrate on linear discriminant analysis effect size (LEfSe) in 12-week-old rats. The LEfSe highlights significant differences in bacterial taxa between the (**A**) CN, MPL, and MPH groups and (**B**) between the MPH and MPHB groups; eight per group.

LEfSe analysis identified the taxa with the most significant abundance differences among the groups. As revealed in Figure 5A, *Bifidobacterium pseudolongum* and its corresponding genus, family, order, class, and phylum were more abundant in the MPL group (LDA > 4). Additionally, *Schaedlerella* and its associated genus were significantly enhanced in the MPHB group (Figure 5B).

**Figure 6:**
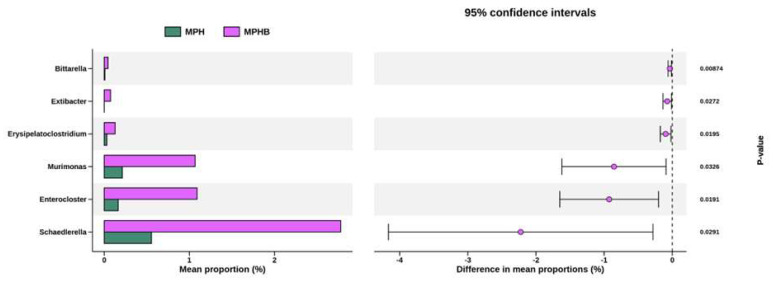
Differentially abundant taxa between the MPH and MPHB groups as identified by STAMP analysis with 95% confidence intervals. Eight per group.

To identify genus-level taxa altered by sodium butyrate treatment, we used the two-sided Welch’s *t*-test in STAMP to determine statistical differences (Figure 6). Compared to the MPH group, sodium butyrate treatment led to significant increases in genera *Bittarella*, *Extibacter*, *Erysipelatoclostridium*, *Murimonas*, *Enterocloster*, and *Schaedierella* (Figure 6).

### 3.5. Plasma SCFAs and SCFA Receptors

**Figure 7:**
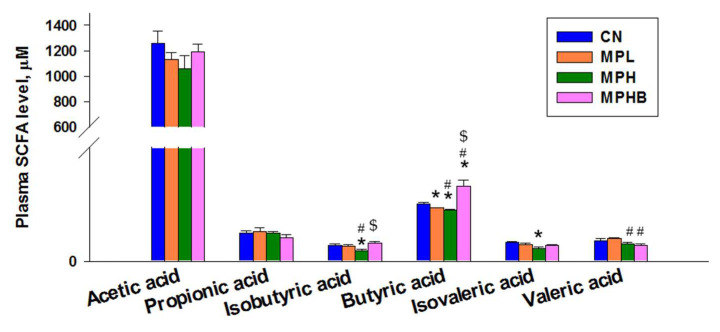
Effect of microplastics and sodium butyrate on plasma concentrations of short-chain fatty acids in 12-week-old rats. Eight per group; * *p* < 0.05 vs. CN; # *p* < 0.05 vs. MPL; $ *p* < 0.05 vs. MPH, indicating that group differences reached statistical significance.

Figure 7 presents the effects of MP and sodium butyrate on plasma SCFA concentrations. Low-dose MP exposure in the MPL group resulted in a significant reduction in plasma butyric acid levels, suggesting that even a low dose of MP can disrupt normal SCFA production. In contrast, high-dose MP exposure in the MPH group led to a decrease in both isovaleric acid and butyric acid levels, indicating a more pronounced impact on SCFA metabolism with increased MP exposure. Notably, in the MPH group, plasma concentrations of isobutyric acid and butyric acid were significantly lower compared with the MPHB group, which received sodium butyrate treatment. This suggests the potential therapeutic effect of sodium butyrate in restoring SCFA balance and mitigating the metabolic disturbances induced by high-dose MP exposure.

**Figure 8:**
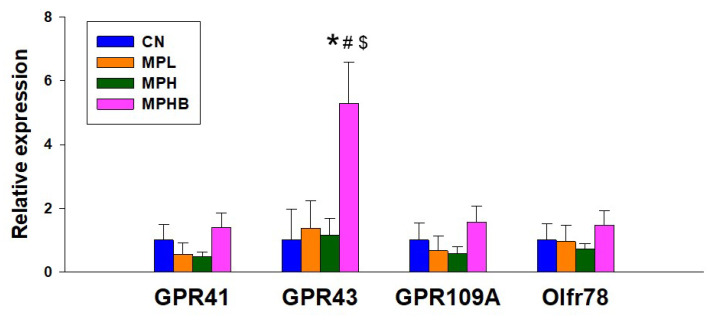
Effect of microplastics and sodium butyrate on renal mRNA expression of SCFA receptors in 12-week-old rats. This figure illustrates the renal levels of GPR41, GPR43, GPR109A, and Olfr78 at 12 weeks of age. Eight per group; * *p* < 0.05 vs. CN; # *p* < 0.05 vs. MPL; $ *p* < 0.05 vs. MPH, indicating that group differences reached statistical significance.

We next assessed mRNA expression of SCFA receptors in the kidneys, as SCFAs are known to work together with these receptors to control BP and contribute to renal function [23]. As shown in Figure 8, renal expression of GPR41, GPR109A, and Olfr78 did not differ among the four groups, suggesting that these receptors might not be directly affected by MP exposure or sodium butyrate treatment in the context of this study. However, a remarkable finding was that renal GPR43 expression was significantly higher in the MPHB group compared with the other groups following sodium butyrate treatment. This increase in GPR43 expression suggests that sodium butyrate may enhance the renal response to SCFAs, potentially contributing to a protective effect against MP-induced hypertension and kidney damage.

## 4. Discussion

**Figure 9:**
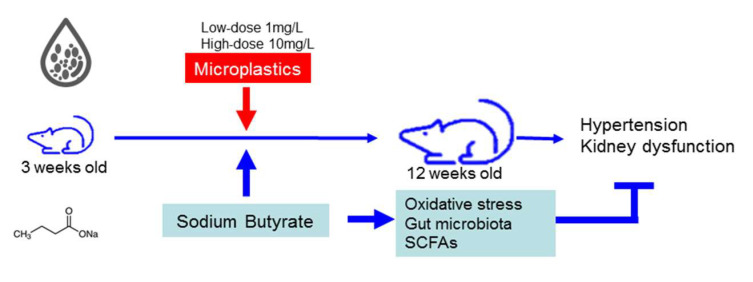
Schematic diagram illustrating the therapeutic effects of sodium butyrate in young rats exposed to microplastics.

This study is the first to demonstrate that sodium butyrate treatment alleviates MP-induced hypertension and kidney disease in young rats, likely through reduced oxidative stress, altered gut microbiota, increased butyric acid, and enhanced renal GPR43 expression. A schematic summarizing the hypothesis and main findings is presented in Figure 9.

Our key findings are as follows: (1) MP exposure leads to kidney function impairment, with an increase in BP observed only at higher doses, suggesting a dose-dependent effect of MPs on kidney health and BP regulation; (2) Sodium butyrate treatment alleviated hypertension and kidney damage associated with high-dose MP exposure, highlighting its potential therapeutic role in mitigating the adverse effects of environmental stressors; (3) MP-induced hypertension and kidney disease are associated with oxidative stress, as evidenced by increased 8-OHdG expression, which works as a marker of DNA damage and underscores the oxidative mechanisms involved in MP-related organ damage; (4) MP exposure induces chronic inflammation through the increased production of both proinflammatory and anti-inflammatory cytokines, indicating a complex immune response that may contribute to kidney damage and BP dysregulation; and (5) The protective effects of sodium butyrate against MP-induced hypertension and kidney dysfunction are linked to elevated plasma butyric acid levels and enhanced renal expression of GPR43. Collectively, these findings suggest that sodium butyrate could be a promising therapeutic approach to mitigate the harmful effects of MP exposure in this animal model.

The associations between environmental plastic pollution, kidney disease, and hypertension have been well documented in various studies [1,2,3,4], but the underlying mechanisms, especially in children, remain poorly understood. Our findings show that young rats exposed to MP exhibited elevated BP and reduced creatinine clearance, suggesting that early MP exposure may signal the onset of hypertension and kidney disease in developing animals.

Building on prior investigations that has suggested the renoprotective and BP-lowering effects of butyrate [24,25], our study affords the first evidence that sodium butyrate treatment improves hypertension and kidney damage in young rats exposed to high-dose MP. This finding is significant, as it suggests that sodium butyrate may serve as a potential therapeutic intervention to counteract the adverse effects of environmental pollutants like MPs on kidney health and cardiovascular function in the developing organism. Our results highlight the importance of considering early-life exposure to microplastic pollutants and exploring protective strategies like sodium butyrate to reduce long-term health risks. They also emphasize the need for further investigation into the mechanisms linking microplastic pollution to hypertension and kidney disease, as well as the potential role of early interventions.

Former work highlights that MP bioaccumulation and its toxic effects on kidneys may be closely linked to oxidative stress [8]. Our findings further support this hypothesis, showing that high-dose MP exposure significantly increases renal 8-OHdG expression, a marker of oxidative DNA damage, indicating a dose-dependent relationship between MP exposure and oxidative damage in the kidneys. Elevated levels of 8-OHdG in the kidneys serve as a key indicator of the cellular damage and dysfunction that results from oxidative stress, which is commonly implicated in the pathogenesis of kidney disease and hypertension. This correlation emphasizes oxidative stress as a principal pathogenic mechanism through which MP exposure contributes to kidney damage and BP dysregulation.

Moreover, our study highlights the potential therapeutic benefits of sodium butyrate, an SCFA with known antioxidant properties. Sodium butyrate treatment in young rats exposed to high-dose MP was shown to significantly reduce hypertension, kidney disease, and oxidative damage, suggesting that butyrate’s antioxidant effects may help counteract the harmful consequences of MP exposure. By modulating oxidative stress pathways, sodium butyrate appears to mitigate the cellular damage induced by MPs, offering a promising strategy to alleviate the detrimental actions of environmental pollutants on kidney and cardiovascular health. Furthermore, the findings propose that managing oxidative stress could be a critical therapeutic target in MP-induced kidney disease and hypertension.

Chronic inflammation induces endothelial dysfunction and contributes to hypertension by increasing ROS production through proinflammatory cytokines [33]. These cytokines raise BP by causing structural and myogenic changes, disrupting the balance between vasoconstrictors and vasodilators. We observed that MP exposure significantly increased serum levels of IL-2, IL-6, IL-10, IL-17A, and IFN-γ. These findings are consistent with prior studies, which have demonstrated high concentrations of pro-inflammatory cytokines such as IL-6, IL-17, and IFN-γ, as well as the anti-inflammatory cytokine IL-10, in both hypertensive patients and animal models [33,34]. Nevertheless, no significant differences were found between MP-exposed rats treated with or without sodium butyrate treatment, suggesting that anti-inflammatory effects may not be the primary mechanism underlying the observed protective effects.

Although previous studies have suggested that MPs may influence the gut microbiota, the findings have been highly variable. This variability is likely attributable to differences in the animal models employed and the diverse nature of MP exposures [35]. In contrast, our data revealed that MP exposure, whether at low or high doses, had no effect on the richness or evenness of the gut microbiota. Furthermore, the MPH group exhibited no differences in β-diversity compared to the MPL and MPHB groups. We observed that *Bifidobacterium pseudolongum* abundance increased following MP exposure. *Bifidobacterium* is a well-known probiotic with various health benefits, including its use in treating hypertension and kidney disease [36]. Previous research has reported both increased and decreased abundance of this microorganism in association with hypertension in humans and animal models [37]. The abundance of *Bifidobacterium* can either increase or decrease in hypertension, and its variation in response to MPs may represent a cause, consequence, or adaptation—an area that requires further investigation.

However, the protective actions of sodium butyrate treatment may be attributed to its impact on the gut microbiota and microbial metabolites. We observed that sodium butyrate treatment increased the abundance of butyrate-producing bacteria, such as *Enterocloster* and *Schaedlerella* [38,39,40], as well as butyric acid concentrations. Butyrate can interact with SCFA receptors to regulate BP [23]. In our study, sodium butyrate treatment increased renal expression of GPR43, which is known to reduce BP through vasodilation [23]. These findings align with prior research indicating that butyrate may help prevent hypertension and kidney disease by modulating SCFA receptors [25,41]. However, the specific contributions of butyrate-producing bacteria to the protective effects of sodium butyrate against hypertension and kidney disease remain poorly understood.

Our study has some limitations that should be considered when interpreting the results. Firstly, while our findings suggest that MP exposure induces dose-dependent oxidative damage and hypertension, further research is required to explore the mechanisms of oxidative stress and identify molecular targets for managing MP-related hypertension and kidney disease. Understanding these pathways could lead to more precise therapeutic strategies for mitigating the health impacts of environmental plastic pollution. Secondly, while we demonstrated that sodium butyrate has protective effects against hypertension and kidney disease caused by MP exposure in this rat model, further research is necessary to confirm its clinical relevance in humans and explore its efficacy across diverse animal models. One limitation of this study is the absence of a control + sodium butyrate group. Although sodium butyrate supplementation is generally considered safe and is commonly used as a dietary supplement for human health benefits, its potential long-term adverse effects in young rats remain to be clarified. Thirdly, our study primarily focused on sodium butyrate, a single SCFA, highlighting its protective effects. However, the impact of other SCFAs and microbial metabolites remains unexplored. Expanding research to include these metabolites could offer a more comprehensive understanding of how gut-derived metabolites influence health outcomes following MP exposure. While GPR43 mRNA levels indicate the potential for SCFA receptor expression, they do not guarantee receptor activation or downstream signaling. Therefore, further studies, such as protein expression analysis or receptor activation assays, are necessary to directly assess SCFA receptor function. Furthermore, kidney injury was assessed using CCr as an indicator of kidney function, while renal histopathology was not analyzed in this study. Although our previous research has shown that the severity of glomerular and tubulointerstitial injuries correlates with reduced kidney function in animal models of chronic kidney disease [42], it is important to consider the potential impact of MP on the histopathological aspects of kidney damage. Lastly, our study did not include female rats, leaving the potential for sex differences in MP exposure effects and butyrate bioactivity unexplored. Investigating the potential differences in how male and female rats respond to MP exposure and sodium butyrate treatment would be a valuable avenue for future research.

## 5. Conclusions

In summary, our study demonstrated that sodium butyrate treatment mitigates hypertension and kidney dysfunction induced by MP exposure in a young rat model. The findings highlight the potential therapeutic role of reducing oxidative stress, modulating gut microbiota and its metabolites, and activating SCFA receptors in addressing MP exposure-related hypertension and kidney dysfunction. Further research is needed to explore these mechanisms more thoroughly and assess their relevance for human translation.

## Figures and Tables

**Table 1:** Effect of microplastics and sodium butyrate on weighs and blood pressure at age 12 weeks.

Group	CN	MPL	MPH	MPHB
Body Weight (g)	527 ± 16	409 ± 16 *	461 ± 5 *#	468 ± 17 *#
Left Kidney Weight (KW) (g)	2.24 ± 0.07	1.91 ± 0.1 *	1.87 ± 0.04 *	1.97 ± 0.04 *
Left KW/100 g BW	0.43 ± 0.01	0.47 ± 0.02	0.41 ± 0.01 #	0.42 ± 0.01
Systolic BP (mmHg)	133 ± 1	136 ± 1	151 ± 2 *#	138 ± 1 $
Diastolic BP (mmHg)	88 ± 1	93 ± 2	103 ± 3 *#	97 ± 1 $
Mean Arterial Pressure (mmHg)	103 ± 1	107 ± 2	119 ± 2 *#	110 ± 1 $

CN: control rats; MPL: 1 mg/L microplastics; MPH: 10 mg/L microplastics; MPSB: 10 mg/L microplastics + sodium butyrate. Eight per group; * *p* < 0.05 vs. CN; # *p* < 0.05 vs. MPL; $ *p* < 0.05 vs. MPH, indicating that group differences reached statistical significance.

## Data Availability

Data are contained within the article.
